# Incidence of ADL Disability in Older Persons, Physical Activities as a Protective Factor and the Need for Informal and Formal Care – Results from the SNAC-N Project

**DOI:** 10.1371/journal.pone.0138901

**Published:** 2015-09-25

**Authors:** Britt-Marie Sjölund, Anders Wimo, Maria Engström, Eva von Strauss

**Affiliations:** 1 Aging Research Center (ARC), Department of Neurobiology, Care Sciences and Society (NVS), Karolinska Institutet and Stockholm University, Stockholm, Sweden; 2 Faculty of Health and Occupational Studies, Department of Health and Caring Sciences, University of Gävle, Gävle, Sweden; 3 Division of Neurogeriatrics, Department of Neurobiology, Care Sciences and Society (NVS), Karolinska Institutet, Stockholm, Sweden; 4 Department of Public Health and Caring Sciences, Uppsala University, Uppsala, Sweden; 5 The Swedish Red Cross University College, Stockholm, Sweden; University of Naples Federico II, ITALY

## Abstract

**Background:**

The aim of the study was to examine 1) the incidence of disability in Activities of Daily Living (ADL), in persons 78 years and older 2) explore whether being physical active earlier is a significant predictor of being disability free at follow-up and 3) describe the amount of informal and formal care in relation to ADL-disability.

**Methods:**

Data were used from a longitudinal community-based study in Nordanstig (SNAC-N), a part of the Swedish National Study on Aging and Care (SNAC). To study objectives 1) and 2) all ADL-independent participants at baseline (N = 307) were included; for objective 3) all participants 78 years and older were included (N = 316). Data were collected at baseline and at 3- and 6-year follow-ups. ADL-disability was defined as a need for assistance in one or more activities. Informal and formal care were measured using the Resource utilization in Dementia (RUD)-instrument.

**Results:**

The incidence rates for men were similar in the age groups 78-81and 84 years and older, 42.3 vs. 42.5/1000 person-years. For women the incidence rate for ADL-disability increased significantly from the age group 78–81 to the age group 84 years and older, 20.8 vs.118.3/1000 person-years. In the age group 78–81 years, being physically active earlier (aOR 6.2) and during the past 12 month (aOR 2.9) were both significant preventive factors for ADL-disability. Both informal and formal care increased with ADL-disability and the amount of informal care was greater than formal care. The incidence rate for ADL-disability increases with age for women and being physically active is a protective factor for ADL-disability.

**Conclusion:**

The incidence rate for ADL-disability increases with age for women, and being physical active is a protective factor for ADL-disability.

## Introduction

Human life expectancy is increasing worldwide [1, 2]. As we live longer, it is interesting for both society and the individual to explore these added years in terms of functional capacity and morbidity. The concepts of expansion, postponement and compression of morbidity/functional capacity are often used in these discussions [3–5]. Will we live with or without disability in activities of daily living (ADL)? Living these extra years with disability will lead to reduced quality of life for the individual as well as to higher costs for society. One Swedish study—using data from a population-based study of persons 60 years and older, and examining how costs vary by level of functioning and with the presence of a brain disorder—found that it was function rather than diagnosis that contributed to increased costs [6]. For the individual, lower quality of life has been found among older persons with ADL-disability irrespective of living situation, that is, at home or in residential care [7, 8].

Most studies are cross sectional and focus on the prevalence of ADL-disability [9–11]. However, of particular interest is how and when ADL-disability starts in a person’s life, and from that perspective, the incidence of ADL-disability rather than the prevalence should be in focus. The literature in this area is sparse. Some studies have found the incidence of ADL-disability to be higher in women than in men. A study from The Netherlands conducted between1990 and 1999, examined 1129 persons 55 years and older, who were ADL-disability free at baseline. At a 6-year follow-up, 26.7% showed ADL-disability. The incidence of ADL-disability was higher in women (33.2%) than in men (19.7%). Women had also a higher proportion of severe disability [12]. The incidence rate for ADL-disability was also higher in women than in men in a study from Brazil. They examined persons 60 years and older who had no difficulties in ADL at baseline in 2000 and, again, at follow-up 6 years later. The incidence for women were 42.4/1000 person-years and for men 17.5/1000 person-years [13]. The higher prevalence of disability in women can be explained by a combination of higher incidence and longer duration resulting from lower rates of recovery and mortality compared with men [14], while men have a lower life expectancy that may be explained by biological and clinical factors. A review study from the US, using data from different studies, found that men had higher mortality due to coronary heart disease, hypertension, diabetes, and cancer than women did [15]. For the individual as well as the society preventive factors for ADL-disability is at interest. Physical activity has been showed to be a protective factor for many health problems [16–18], and to be associated with less ADL-disability in old age [19–21]. A study from Italy using data from a population-based study in 1998–2003 of persons 65 years and older found at follow-up after 3-years that a higher level of physical activity was a protective factor for development of ADL-disability [19]. An intervention study from US of 400 persons 70 years and older who had deficit in mobility found after 12 month that the participants in the physical intervention-group had significant better improvement in physical functioning than the control-group [20]. A cohort study from US examined 787 persons living in senior housing facilities with no ADL-disability at baseline. After 2.6 years they found that persons who reported 2.33 hours of physical activity per week had 16% less ADL-disability compared with persons that reported no physical activity [22, 23]. Physical activity has also shown to improve ADL in persons with dementia [24]. Based on earlier research we hypothesized that incidence rate of ADL-disability would be higher in women than in men and increase with age, furthermore that being physical active earlier in life could be a preventive factor for ADL-disability in old age.

Care of older persons due to disability involves a complex interaction between formal and informal care resources. Care is also highly dependent on the socioeconomic context, on both the micro- (families) and macro level (how care is financed and organized in a society/country). Besides economic strength on both the micro- and macro- level, traditions, politics and culture also contribute to the complex situation.

The aims of this paper are first to examine the incidence of impaired physical functioning defined as ADL- disability, in relation to gender; second to explore whether being physically active earlier in life and/or during the past 12 month are significant predictors of being disability free at follow-up. Due to the relationship between ADL-disability and resource use and costs, a third aim is to describe the amounts of informal and formal care in relation to levels of ADL-disability.

## Materials and Methods

### Study design

This study was based on data from the Swedish National Study on Aging and Care in Nordanstig (SNAC-N). SNAC-N is a longitudinal individual population-based ongoing study being conducted in the municipality of Nordanstig in Sweden, which is a rural area in the northern Sweden and had, at the time for the baseline collection, approximately 10 000 inhabitants. This coastal district has no city or central areas–instead there are several small villages covering an area of 1 380 square kilometers. SNAC-N is one of four geographical areas in focus in a larger national study promoted by the Swedish Ministry of Health and Social Affair [25].

### Study population

In the present study, we used data from participants who were independent in ADL and 78, 81, 84, 87, 90, 93, 96, and 99+ years of age at baseline (N = 307) to study incidence rates. For our analysis of informal and formal care, we used data from all participants who were 78 years and older at baseline and living at home (N = 316).

### Ethics

The study was approved by the Ethics Committee of the Karolinska Institutet and the Regional Ethical Review Board in Stockholm.

### Data collection

Baseline data were collected from March 2001 to March 2003 and data for the 1^st^ follow-up three years later and the 2^nd^ follow-up six years later. Data were gathered through interviews and clinical examinations using standardized protocols administered by a trained registered nurse and a licensed practical nurse. A physical examination was also carried out by a physician. Information from a proxy interview was used when the participant was unable to answer or was diagnosed with dementia.

### Study variables

#### Sociodemographic factors and mortality data

Sociodemographic factors were age, gender and education. Mortality data were gathered regularly from the Swedish National Death Certificate Register.

#### ADL

Basic ADL was measured by interviewing and observing the participants and using the hierarchical scale KATZ index of ADL to assess dependency in basic activities [26]. Disability was defined as a need for assistance with one or more activities. We used a modified version that assess dependency in five basic activities: bathing, dressing, going to the toilet, transferring and feeding [13, 27].

#### Physical activity

At baseline, study participants were asked whether they had engaged in regular light exercise (walking, golf, short-distance cycling) 1) earlier in life and 2) during the past 12 months. Response alternatives were: every day, several times/ week, 2–3 times/month, less or never.

#### Cognition

Cognition was measured using the Mini-Mental State Examination (MMSE), a commonly used instrument scoring between 0–30 points, where 30 points represents no impairment [28].

#### Formal and informal care

Parts of the Resource Utilization in Dementia (RUD) instrument was used to calculate the amount of formal and informal care at baseline and follow-ups in the participant´s residence [29]. In the present study, hospital care, visits to clinics, etc., are not included. Formal care by home aides is the care provided by the municipality, and informal care is that provided by relatives, neighbors and friends. The validity and reliability of the instrument has been investigated for persons living in their regular homes and in residential care settings. Results have shown that interviews concerning the amount of help with ADL and Instrumental activities of daily living (IADL), are a valid and reliable substitute for observations [29, 30]. Data were gathered by interviewing the participants or proxy if the participant could not give reliable information. Owing to the type of care, the time frame for questions about IADL was the past month, while for basic ADLs it was the past week. Data on IADL and ADL for both formal and informal care are presented as hours per month.

### Statistical analysis

Age- and gender- specific incidence rates were calculated at both the 3-year and 6-year follow-up, using as the numerator all cases with a diagnosis of ADL-disability (needing assistance in one or more or two or more ADL-activities), and as the denominator, the examined population. Age- and gender- specific incidence rates were calculated as the number of new cases divided by the person-years at risk. The 95% confidence intervals (CI) were based on the Poisson distributions. Person-years for non-disabled subjects were calculated as the time between baseline examination and the follow-up examinations. For the ADL-disabled subjects, half this time was assumed, due to the uncertainty of disability onset. Furthermore, a subject who developed any type of disability was considered to no longer be at risk. The incidence rate was calculated in two separate analysis, needing assistance in one or more (ADL1+), or two or more (ADL2+) ADL-activities. Logistic regression analyses were used to estimate the association between physical activity and ADL-disability adjusted for gender and cognition. The results are presented as adjusted odds ratios (aOR), and 95% CIs. Mean values for the number of hours per month of informal and formal care that the persons received were calculated using univariate analyses of variance (One-Way ANOVA).The IBM SPSS Statistics version 20.0 for Windows (IBM SPSS Inc., Chicago, IL) was used for modeling analyses and statistical tests

## Results

**Fig 1:**
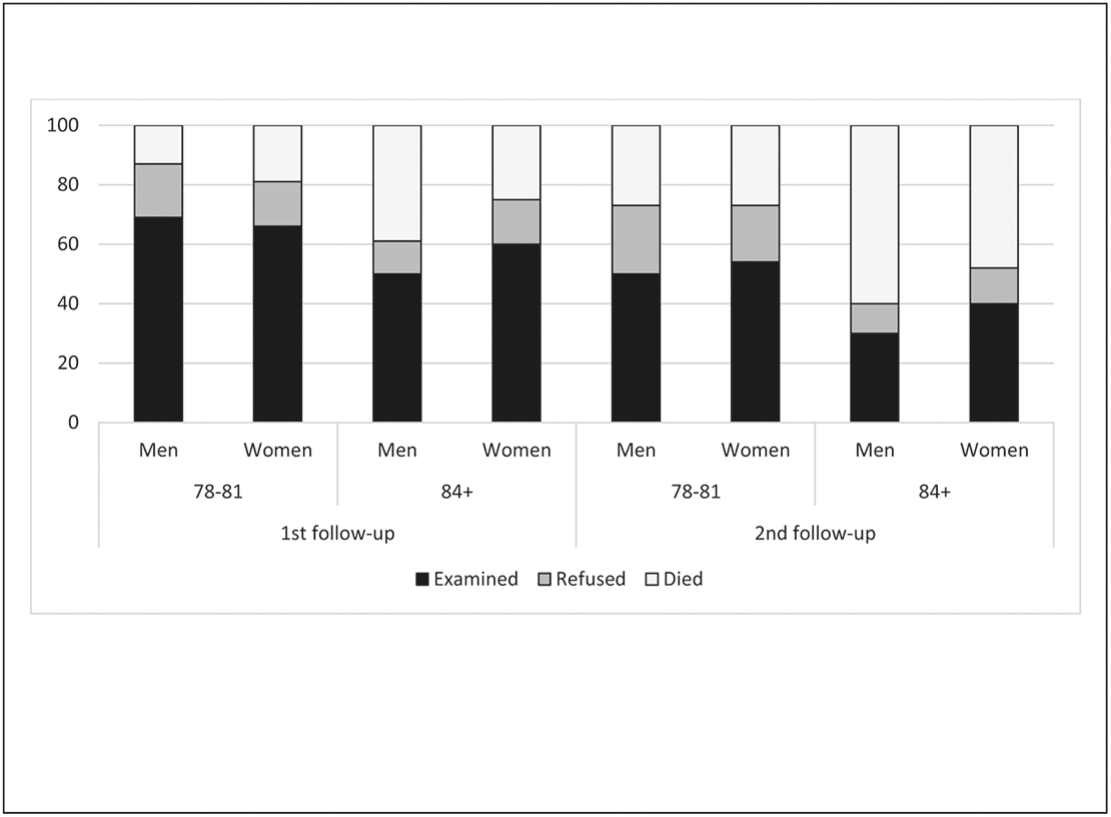
Percentage of participants and drop-outs by causes in the cohort of non-disabled subjects from baseline to 1^st^ and 2^nd^ follow-up. Distribution by age and gender.

The study population consisted of 307 participants, of whom 57% were women. The mean age was 83.2 (SD 4.5) for men and 83.0 (SD 4.5) for women. At the 2^nd^ follow-up after six years, 135 (43.4%) were re-examined, of those 135 participants 18 were not examined at the 1^st^ follow-up (refused or missing). Between baseline and the 2^nd^ follow-up 40.0% of the participants had died, more men than women (44.7% vs. 36.6%). The study population was divided into two age groups, one in the age of 78 and 81 year at baseline (n = 155) and the second 84 years and older at baseline (n = 152). At the 2^nd^ follow-up, 60.0% of the men and 47.6% of the women had died (non-significant) (Fig 1).

### ADL-disability

After three years 10.6% had become ADL-disabled (needing assistance with one or more activities) and after six years 28.1%. There was a non-significant tendency for more men than women in the age group 78–81 to become ADL-disabled, at both the 1^st^ and 2^nd^ follow-up. In the age group 84 years and older, more women (57.6%) than men (23.8%) had become ADL-disabled at 2^nd^ follow-up in terms of needing assistance with one or more activities (p<0.05). Both at 1^st^ and 2^nd^ follow-up and needing assistance with one or more activities (ADL 1+), or two or more (ADL 2+), women had become more ADL-disabled in the age group 84 years and older than women in the age group 78–81 (p<0.05). There were no significant differences in ADL-disability between men in the two age groups (Table 1).

**Table 1:** Number of incident cases of ADL-disability at 1^st^ follow-up (after three years), and at 2^nd^ follow-up (after six years), separately for needing assistance with one or more activities (ADL 1+) and needing assistance with two or more activities (ADL 2+), in the cohort of non-disabled subjects at baseline. Distribution by age and gender.

	1^st^ follow-up (n = 188)		2^nd^ follow-up (n = 135)	
	ADL 1+	ADL 2+	ADL 1+	ADL 2+
	(%)	(%)	(%)	(%)
**Men**				
78–81	3 (7.0)	2 (4.7)	7 (22.6)	4 (12.9)
84+	3 (8.6)	2 (5.7)	5 (23.8)	5 (23.8)
Total	6 (7.7)	4 (5.1)	12 (23.1)	9 (17.3)
**Women**				
78–81	3 (4.9)	2 (3.3)	7 (14.0)	3 (6.0)
84+	11 (22.4)	9 (18.4)	19 (57.6)	14 (42.4)
Total	14 (12.7)	11 (10.0)	26 (31.3)	17 (20.5)
**All**	20 (10.6)	15 (8.0)	38 (28.1)	26 (19.3)

The overall incidence rate for ADL-disability was higher for women, 59.3/1000 person-years, than for men 42.4/1000 person-years. The incidence rate for disability increased with age especially for women (Table 2). The incidence rate was also higher for women than for men in the age group 84 years and older, and for receiving assistance both with one or more (ADL 1+) and with two or more (ADL 2+) activities. The incidence rates for men were almost identical in the age group 78–81 years and the age group 84 years and older, 42.3 vs. 42.5/1000 person-years. For women, the incidence rate for ADL-disability increased significantly from the age group 78–81 years to the age group 84 years and older both for ADL1+ (20.8 vs. 118.3/1000 person-year) and ADL 2+ (8.5 vs. 81.5/1000 person-year).

**Table 2:** Needing assistance with one or more activities (ADL 1+) and needing assistance with two or more activities (ADL 2+): person-years at risk (py), number of cases (n), and incidence rates (IR) per 1,000 person-years by age and gender in the cohort of non-disabled subjects at baseline.

	ADL 1+			ADL 2+		
Age groups	py	n	*IR*	py	n	*IR*
			*95 % CI*			*95% CI*
**Men**						
78–81	212.8	9	42.3 (19.3–80.3)	220.3	6	27.2 (10.0–59.3)
84+	164.6	7	42.5 (17.1–87.6)	170.6	7	41.0 (16.5–84.5)
All	377.4	16	42.4 (24.2–68.8)	390.9	13	33.3 (17.7–56.9)
**Women**						
78–81	336.9	7	20.8 (8.4–42.8)	352.6	3	8.5 (1.8–24.9)
84+	219.8	26	118.3 (77.3–173.3)	233.1	19	81.5 (49.1–127.3)
All	556.7	33	59.3 (40.8–83.2)	585.7	22	37.6 (23.5–56.9)
**All**						
78–81	549.7	16	29.1 (16.6–47.3)	572.9	9	15.7 (7.2–29.8)
84+	384.4	33	85.8 (59.1–120.6)	403.7	26	64.4 (42.1–94.4)
All	934.1	49	52.5 (38.8–69.4)	976.6	35	35.8 (25.0–49.8)

### Physical activity as a preventive factor

Of the 155 persons in the age group 78–81 years, 126 answered the question about physical activity, and in the age group of 84 years and older, 84 of 152 answered the question. Of those who answered the question, 2% in the age group 78–81 and 3,8% in the age group 84 years and older reported never having been physically active earlier in life, and 7.9% and 10.7% respectively, reported never having been physically active during the past 12 months. In the age group 78–81 years, there was a significant association between being physically active during the past 12 month prior to baseline examination, aOR 2.9 (1.3–6.2) and earlier in life, aOR 6.2 (1.3–29.3) and being non ADL-disabled at the follow-ups. No association was found between physical activity and ADL-disability in persons 84 years and older, see Table 3.

**Table 3:** Adjusted Odds Ratio (aOR) and 95% Confidence Intervals (CI) for the association between being ADL-disability free at follow-ups and physical activity (earlier in life or the past 12 month).

	Physical activity	
Age group	Earlier in life*	The past 12 month
78–81 (n = 126)	6.2 (1.3–29.3)	2.9 (1.3–6.2)
84+ (n = 84)	0.8 (0.5–1.6)	0.9 (0.6–1.6)

Adjusted for gender and cognition.

*Missing data for 26 persons in the age group 78–81 and 4 persons in the age group 84+.

### Informal and formal care

**Fig 2:**
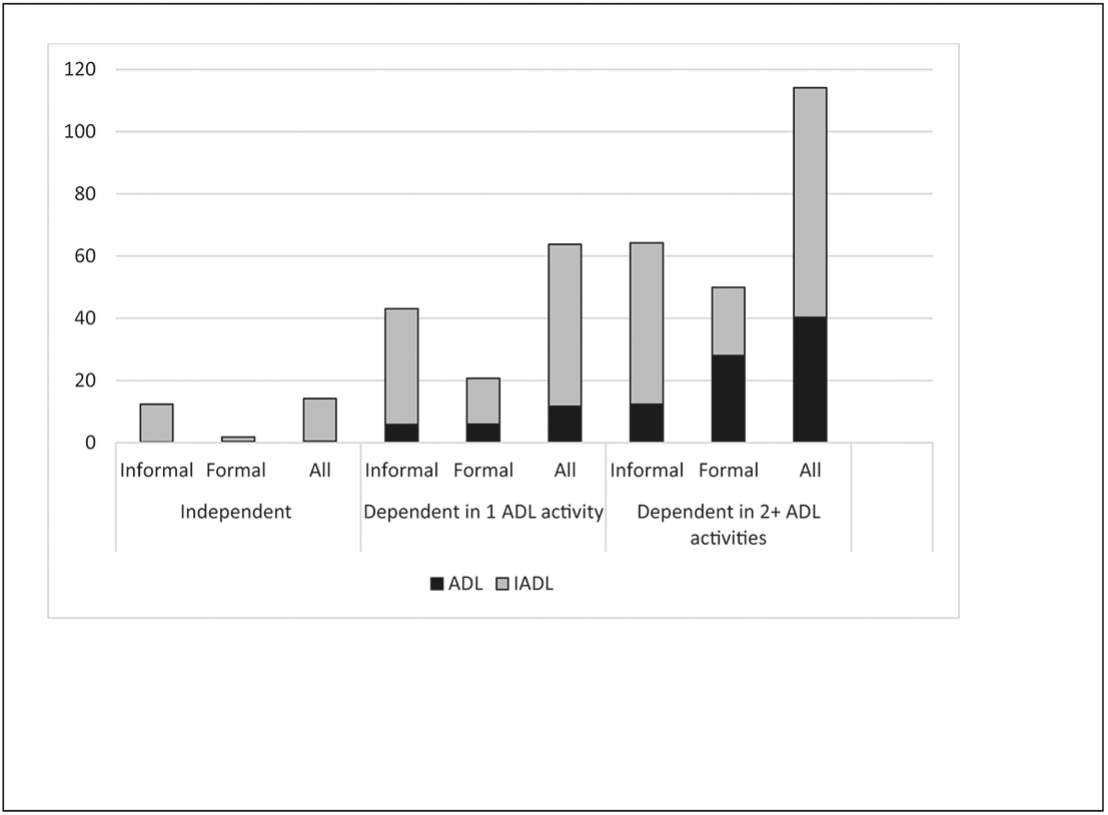
The amount of informal and formal care (mean hours per month). Distribution by ADL-disability (N = 316).

In the study population of both ADL-disabled and non-ADL-disabled persons, the mean hours per month was for the informal care 15.7 and for the formal care 4.6 hours/month. Among the persons still living at home at the follow-ups, the amount of both informal and formal care increased with the number of ADL activities the persons were required help with, and the differences in amount of care between the ADL groups were significant (p<0.05). When the formal and informal care is divided into assistance in IADL and ADL, the analysis showed that the informal care mainly consisted of assistance with IADL and the formal care of assistance with both IADL and ADL. Between baseline and the two follow-ups, 34 persons had moved to residential care. Their ADL-dependency varied between 0–5. Thirty persons were living alone at home before they moved and only four persons were living together with their wife and were dependent in 4 or 5 ADL-activities (Fig 2).

## Discussion

The aims of the present study were to examine the incidence of impaired physical functioning defined as ADL disability, to explore whether being physical active earlier is a significant predictor of being disability free at follow-up and to describe the amount of informal and formal care received in relation to ADL-disability. The main findings from the study can be summarized as follows:

1. The incidence rates for men were almost identical in the age group 78–81 and the age group 84 years and older 42.3 vs. 42.5/1000 person-years. For women the incidence rate for ADL-disability increased significantly from the age group 78–81 to the age group 84 years and older, both for ADL1+ (20.8 vs. 118.3/1000 person-year) and ADL 2+ (8.5 vs. 81.5/1000 person-year).
2. In the age group 78–81 years, being physically active earlier (aOR 6.2) and during the past 12 month (aOR 2.9) were both significant preventive factors for ADL-disability.
3. The amount of both informal and formal care increased with ADL-disability, and the amount of informal care was greater than that of formal care

### Incident of ADL-disability

We found that, of the non-disabled population 78 years and older at baseline, 10.6% had become AD-disabled after three years and 28.1% after six years. This is almost the same result as in the study from the Netherlands [12].The incident of ADL-disability after six years was 26.7% in their study of persons 55 years and older, with a higher incidence in women than in men. In addition to focusing on different age groups, the present study and the Dutch study used different instruments to collect disability data. The Dutch study used an 8-item questionnaire to measure disability. This may explain why the prevalence’s are almost the same despite the different age groups.

The overall incidence rate for ADL-disability was higher for women, 59.3/1000 person-years, than for men 42.4/1000 person-years. Higher incidence rate in women than in men was found in the study from Brazil also. The incidence rate was lower in the Brazilian study due to the younger study population, 60 years and older [13]. Dividing the present study population into two age groups showed that the IR for disability in the age group 84 years and older was significantly higher for women than that for men, in both ADL1+ (118.3 vs. 42.5/1000 person-years) and ADL2+ (81.5 vs. 41.0/1000 person-years). The IR for disability for men in the two age groups was unchanged, while it increased a greater deal for women in the age group 84 years and older. The higher IR in disability and proportion of new incidence cases for women 84 years and older compared with men in the same age group, and the higher mortality rate after six years among men (60.0%) than among women (47.6%) older than 84 years may explain the higher prevalence of ADL-disability in older women than in older men. Similar results have been found in other studies [12, 31, 32].

### Physical activity

In the age group 78–81 years, physical activity earlier in life and/or during the past 12 months prior to baseline examination was shown to be a protective factor for ADL-disability at the follow-ups. In the present study, physical activity referred to activities such as biking, walking in the forest, golfing and other not particularly intensive physical activities. Other studies have also shown that physical activity can reduce disability in old age [22, 33] and that light physical exercise in midlife may reduce the risk for dementia in adulthood [16]. The LIFE Study randomized clinical trial from the United states found that the intervention group with physical activity reduced their disability over 2.6 years among older persons at risk for disability when compared with the control group having an health education program [33]. Physical exercise also improves balance and muscle strength [34] thus reducing the risk for fall injuries that could cause disability [35]. The item “physical activity during the past 12 months” before baseline could be confounded by an ongoing disease that had not yet resulted in disability at baseline, while the item “physical activity earlier in life” seems to give stronger support for the role of physical activity in the prevention of disability. Furthermore, a study by Guralnik et al [36] stated that measures of lower-extremity function in non-disabled persons may predict onset of subsequent disability, and thus identify older adults who may benefit from interventions to prevent the development of severe disability.

### Informal and formal care

The amount of care increased with the number of ADL-activities the person received help with. For persons who were independent in ADL and needed help with IADL-activities, help was mainly informal. When persons were more disabled, the pattern was different. Help with IADL was mostly informal and help with ADL was mostly formal. When persons were disabled in two or more ADL-activities, the amount of formal care was greater than the amount of informal care, mainly due to help with ADL. In the present study, nine persons moved to residential care between the follow-ups. Those persons received help with 0–5 ADL-activities, but all were living alone at home before moving. Of those in the study who were still living at home and were receiving help with two or more ADL-activities, all were living with a spouse. The present study shows that the informal care provided by co-habiting persons strongly contributes to allowing older people to stay at home longer before moving to residential care. Informal care is also important for society as a whole, because it replaces formal care and without it, older persons would probably have to move to residential care earlier. The amount of informal and formal care varies between countries. A study examining community care in 11 different countries in Europe found that formal care was small in Italy, while in the UK it was more than double the European average across all levels of dependency [37]. Another study from Italy reported that the amount of formal care was low and that is was common that disabled older Italians received care from family members and usually from two or more persons [38]. However, informal caregivers have reported a greater burden, isolation and lower perceived health compared to same-age persons not providing such care [39]. Therefore, it is important to support informal caregivers, and to help them provide the care they wish to provide for their relatives. Other studies have also shown that the amount of the informal care is greater than the formal care and that informal caregivers can postpone move to residential care for the care recipient [40–42].

### Limitations

The limitation of the present study may be the size of the study population. Another limitation can be that we have not taken into account whether those who died during follow-up had developed disability as we lack the data. This might have led to an underestimation of disability-incidence. The reason why there were so few people receiving assistance in two or more ADL activities, was probably because the majority of people with disability in two or more ADL activities had moved to residential care. The strengths of the present study is its longitudinal individual population-based design, which is preferable to cross-sectional designs. The data collection team members were almost identical at baseline and the follow-ups, thus minimizing reliability problems and that the study use validated instruments e.g. MMSE, Katz index of ADL, RUD.

## Conclusion

The incidence rate for ADL-disability increases with age for women, and being physical active is a protective factor for ADL-disability.
